# Supplementary material for: Kinetic mechanism of Na+-coupled aspartate transport catalyzed by GltTk
Source: Commun Biol. 2021 Jun 17;4:751. doi: 10.1038/s42003-021-02267-y (PMC8211817; doi:10.1038/s42003-021-02267-y)
Supplement: Supplementary file 2 — Supplementary Information [file 42003_2021_2267_MOESM2_ESM.pdf]

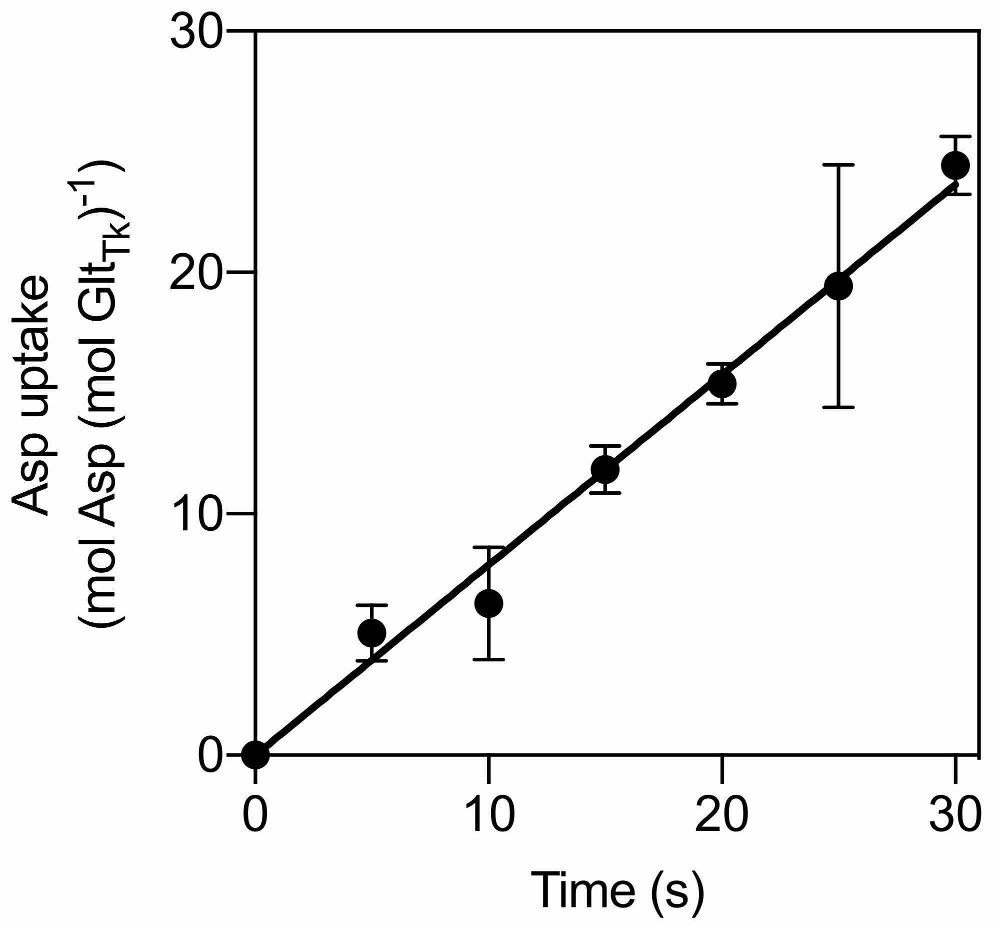

Supplementary Figure 1. Time course of aspartate uptake in proteoliposomes with reconstituted Glt$_{Tk}$. v_{max} conditions were used (300 mM Na^+ and 100 µM L-Aspartate). The experiment was done in triplicate with the error representing the standard deviation. From this result we concluded that the transport rate is constant over the first 30 seconds of the experiment.
