# Supplementary material for: Kinetic mechanism of Na+-coupled aspartate transport catalyzed by GltTk
Source: Commun Biol. 2021 Jun 17;4:751. doi: 10.1038/s42003-021-02267-y (PMC8211817; doi:10.1038/s42003-021-02267-y)
Supplement: Supplementary file 3 — Description of Supplementary Files [file 42003_2021_2267_MOESM3_ESM.pdf]

Description of Additional Supplementary Files

File name: Supplementary Data 1

Description: The source data underlying Figures 1-4 and Tables 1-4.
